# The relationships between HLA class II alleles and antigens with gestational diabetes mellitus: A meta-analysis

**DOI:** 10.1038/srep35005

**Published:** 2016-10-10

**Authors:** Cong-cong Guo, Yi-mei Jin, Kenneth Ka Ho Lee, Guang Yang, Chun-xia Jing, Xuesong Yang

**Affiliations:** 1 Medical College, Jinan University, Guangzhou 510632, China; 2 Key Laboratory for Regenerative Medicine of the Ministry of Education, School of Biomedical Sciences, Chinese University of Hong Kong, Shatin, Hong Kong; 3 Key Laboratory of environmental exposure and health in Guangzhou, Jinan University, Guangzhou, 510632, China

## Abstract

Gestational diabetes mellitus (GDM) is defined as glucose intolerance with onset or first recognition during pregnancy. It is associated with an increased risk of pregnancy complications. Susceptibility to GDM is partly determined by genetics and linked with type 1 diabetes-associated high risk HLA class II genes. However, the evidence for this relationship is still highly controversial. In this study, we assessed the relationship between HLA class II variants and GDM. We performed meta-analysis on all of literatures available in PubMed, Embase, Web of Science and China National Knowledge Infrastructure databases. The odds ratio and 95% confidence interval of each variant were estimated. All statistical analyses were conducted using the Comprehensive Meta Analysis 2.2.064 software. At the allelic analysis, DQB1*02, DQB1*0203, DQB1*0402, DQB1*0602, DRB1*03, DRB1*0301 and DRB1*1302 reached a nominal level of significance, and only DQB1*02, DQB1*0602 and DRB1*1302 were statistically significant after Bonferroni correction. At the serological analysis, none of DQ2, DQ6, DR13 and DR17 was statistically significant following Bonferroni correction although they reached a nominal level of significance. In sum, our meta-analysis demonstrated that there were the associations between HLA class II variants and GDM but more studies are required to elucidate how these variants contribute to GDM susceptibility.

Gestational diabetes mellitus (GDM) is defined as glucose intolerance with onset during pregnancy^1^. The manifestation of GDM is reportedly influenced by age^2^, ethnicity^3^, BMI^4^, and family history of GDM of the pregnant woman^5^. Despite all this information, the pathogenesis of GDM still remains obscure. Since GDM is regarded as a risk factor for developing type 2 diabetes^6^, many investigators have mainly focused on the linkage between GDM and type 2 diabetes. However, Lapolla *et al*.^7^ reported that presentation of pancreatic islet autoantibodies during GDM is predictive for type 1 diabetes development. A number of studies have demonstrated that the circulating immune markers of type 1 diabetes (such as anti-islet cell antibodies and anti-GAD antibodies) are present in the blood of pregnant women with GDM^8^,^9^,^10^,^11^. There is no doubt that we could understand the pathology underlying GDM better, if more genetic risk variants that are shared by type 1 diabetes and GDM were identified. The major type 1 diabetes susceptibility variants are HLA class II genes located on chromosome 6p21, which account for up to 30–50% of the heritability of type 1 diabetes^12^. In this context, it is important to establish whether or not HLA class II alleles are contributory factors of GDM development.

Previous association studies have suggested a role for HLA class II variants in the pathogenesis of GDM. For HLA-DQ alleles, DQB1*02 was reported to be positively associated with GDM in African-American^13^ and Swedish^11^ populations, while DQB1*0602^14^,^15^ and DQB1*0402^16^ were negatively related with GDM in Swedish and Chinese populations, respectively. For HLA-DR variants, DR5^17^ has been found to be negatively associated with GDM in Italian patients, while DRB1*0301^18^ and DRB1*1302^19^ were positively linked with GDM in Chinese patients. However, the associations between these variants and GDM differ from the conclusions drawn in other ethnicities studies. There is the possibility that the relative small sample sizes and varying characteristics of the human population may generate false-positive associations and misinterpretations.

Meta-analysis is a valued method that integrates the findings of multiple investigations, which enhances the statistical power and generate a more definitive conclusion^20^. Hence, in this study, we employed a meta-analytical approach to determine whether there are the associations between HLA class II variants and GDM by analyzing all the published data available in biomedical databases.

## Results

### Features of the publications selected for investigation

**Figure 1:**
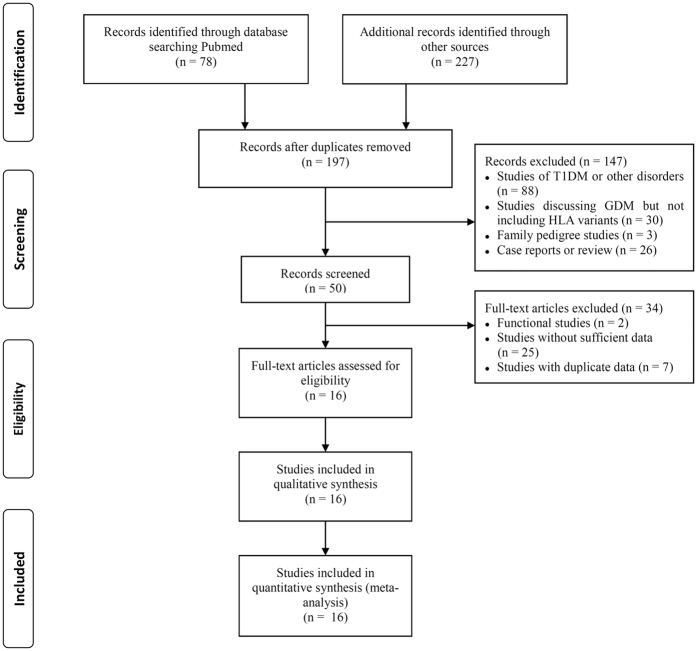
Flow chart showing the literature selection procedure used in this study.

In the preliminary database search, we identified a total of 305 articles, of which 50 publications were potentially relevant to our study. After screening the full-text of the papers, we excluded 34 from the study because they were functional studies or presented duplicate data sets or they did not supply sufficient data about HLA polymorphisms. In our analysis, we finally employed a total of 16 studies^11^,^13^,^15^,^16^,^17^,^18^,^19^,^21^,^22^,^23^,^24^,^25^,^26^,^27^,^28^,^29^ and their characteristic features are summarized in Table 1. The flow of our study is illustrated in Fig. 1. Amongst the 16 studies, we examined the associations between HLA class II variants and GDM in a total of 3122 patients and 3439 control subjects. The number of controls in the each individual studies ranged from 0.37 to 3.07 per case. Based on the Newcastle-Ottawa Quality Assessment Scale (NOS), 3 studies were defined as high quality (all of them scored 7), 12 studies were defined as moderate quality (6 studies scored 6, 5 studies scored 5 and 1 study scored 4), and 1 study was defined as poor quality (scored 2) (Supplementary Table S3).

### Meta-analysis revealed that HLA DQB1 and DRB1 are associated with GDM

**Figure 2:**
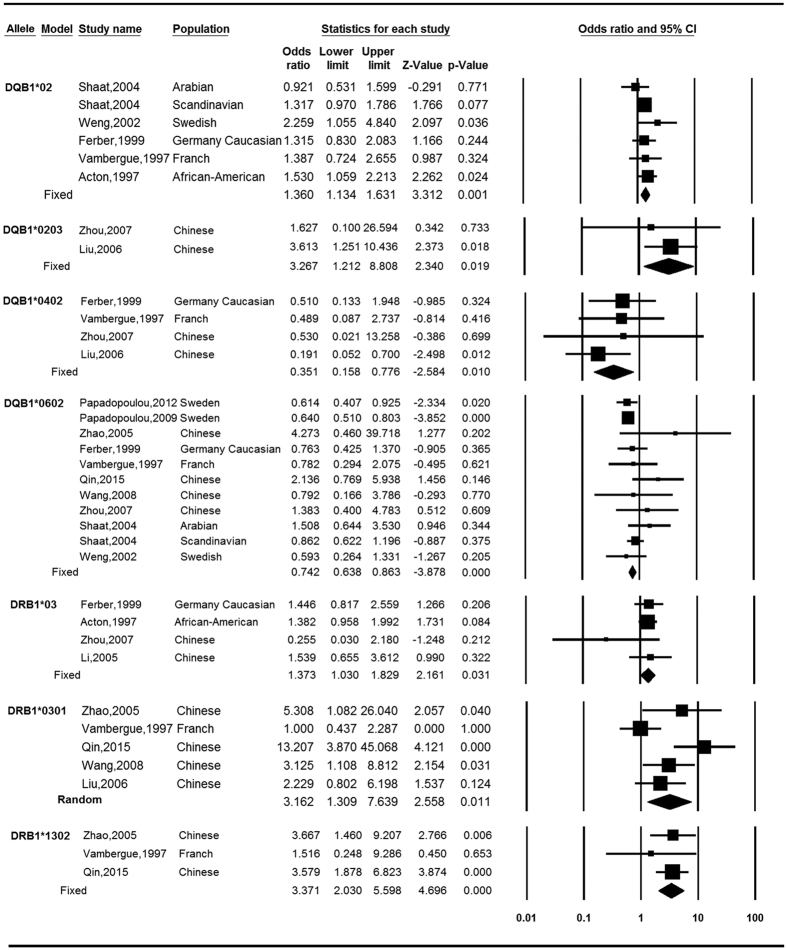
Meta-analysis: forest plots of the associations between HLA alleles and gestational diabetes mellitus.

**Figure 3:**
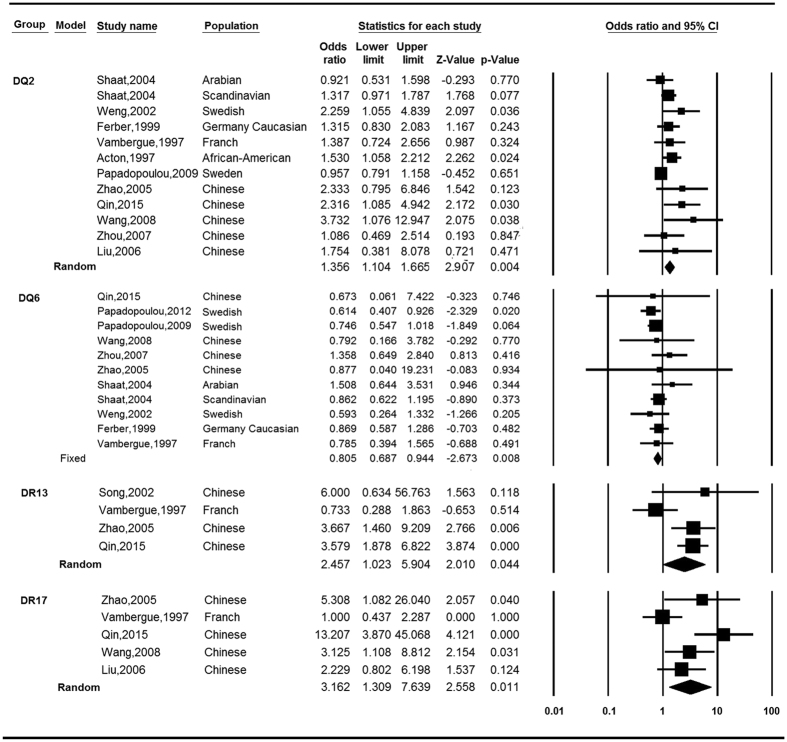
Meta-analysis: forest plots of the associations between HLA serologic groups and gestational diabetes mellitus.

Tables 2 and 3 present number of populations, OR along with 95%CI and *I*^*2*^-statistic for results of meta-analyses. At the allelic level, seven of them reached nominally significant association with GDM (Fig. 2). Specifically, DQB1*02 (OR = 1.36, 95% CI = 1.13–1.63), DQB1*0203 (OR = 3.27, 95% CI = 1.21–8.81), DRB1*03 (OR = 1.37, 95% CI = 1.03–1.83), DRB1*0301 (OR = 3.16, 95% CI = 1.31–7.64) and DRB1*1302 (OR = 3.37, 95% CI = 2.03–5.60) were determined to be associated with increased risk of developing GDM, with etiologic fractions (EFs) of 0.08, 0.05, 0.03, 0.15 and 0.17, respectively. For DQB1*0602 (OR = 0.74, 95% CI = 0.64–0.86) and DQB1*0402 (OR = 0.35, 95%CI = 0.16–0.78), the alleles were associated with a reduced risk of developing GDM, with protective fractions (PFs) of 0.07 and 0.04, respectively. DQB1*02, DRB1*1302 and DQB1*0602 still reached significance after multiple testing correction. No heterogeneity was observed in the analyses besides DRB1*0301 (*P*_*h*_ = 0.012, *I*^2^ = 68.84). At the serological level, we determined there were four groups that demonstrated nominally significant association with GDM (Fig. 3). Negative association was observed for DQ6 (OR = 0.81, 95% CI = 0.69–0.94), with a PF of 0.03. There was no heterogeneity among the 11 populations examined in regards to DQ6 (*P*_*h*_ = 0.743, *I*^2^ = 0). We determined that there were positive associations for DQ2 (OR = 1.36, 95% CI = 1.10–1.67), DR13 (OR = 2.46, 95% CI = 1.02–5.90) and DR17 (OR = 3.16, 95% CI = 1.31–7.64), with EFs of 0.07, 0.07 and 0.15, respectively. In addition, the relationships were heterogeneous amongst the observations of each group (Table 3). Among these four groups, no antigen was still statistically significant after multiple testing correction.

### Publication bias

Begg’s funnel plot and Egger’s test were used to assess for publication bias in our study. We did not detect asymmetry in the shape of the funnel plots for HLA class II variant polymorphisms, which indicate minimal publication bias. The Egger’s tests also showed that the *P*-values were more than 0.05 for all polymorphisms.

## Discussions

Our meta-analysis of 16 association studies, including 3122 GDM cases and 3439 controls, provides by far the most comprehensive assessment about the relevance of GDM of HLA class II variants. The present meta-analysis revealed that four serological groups and seven HLA alleles were nominally associated with GDM. Interestingly, DQB1*02, DQB1*0602 and DRB1*1302 showed a robust association with the development of GDM after Bonferroni correction. DQB1*0602 was determined to act as protective factor against GDM. In contrast, DQB1*02 and DRB1*1302 were found to be risk factors for developing GDM.

The link between DQ6 and GDM was discernible in two Swedish populations and one Chinese population^15^,^19^,^21^. However, studies of other populations have provided conflicting results that both supported and dismissed the DQ6 link with equal frequencies^11^,^18^,^22^,^24^,^25^,^27^,^28^. Using meta-analysis, we have identified that DQ6 is nominally associated with the etiology of GDM (OR = 0.81, 95% CI = 0.69–0.94) and this association remains significant even when we removed any of the publications included in this study. Since the association was no longer significant after the Bonferroni correction, further research is still meaning and noteworthy. Our study also suggests that the role for DQ6 and resistance to GDM is primarily dictated by allele DQB1*0602 (which is a protective factor for type 1 diabetes)^30^. Other alleles such as DQB1*0601, DQB1*0603, DQB1*0604 and DQB1*0605 are not associated with GDM development.

Some HLA class II variants, such as DQB1*02^11^, DQB1*0201^19^, DQB1*0203^16^, DQB1*0301^21^, DQB1*0402^16^, DRB1*01^13^, DRB1*02^13^, DRB1*0301^18^, DRB1*1302^18^ and DR1^26^ have been implicated in GDM development in association studies. However, our meta-analysis result could only confirm the associations for DQB1*02 and DRB1*1302 with GDM. We have found DQB1*02 was in linkage disequilibrium with DRB1*03, and DRB1*03 - DQB1*02 haplotype has been reported to be the most susceptible variant in type 1 diabetes^31^. Furthermore, DRB1*1302 was determined to be in linkage disequilibrium with DQB1*0604, and increased type 1 diabetes risk has been implicated with the DRB1*1302-DQB1*0604 haplotype^32^.

Our meta-analysis elucidated one novel nominally significant variant, DRB1*03, for GDM. Interestingly, DRB1*03 has been reported to be associated with susceptibility to type 1 diabetes^33^,^34^. In fact, all studies involving DRB1*03 have reported an increased rate of occurrence in patients with GDM but these findings were not statistical significant. However, it was not significant after the Bonferroni correction, which suggested no robust association between DRB1*03 and GDM. The relative small samples used in these studies may generate false-negative results and additional polymorphisms might have been identified as in studies with larger sample size.

All of these shared HLA variants highlight the potential immunologic mechanism shared between GDM and type 1 diabetes. Contrary to the insulin resistance of type 2 diabetes, type 1 diabetes is formed as a result of the progressive autoimmune destruction of the pancreatic β–cells^35^. This autoimmune phenomena has been linked with pregnant women with GDM^36^,^37^. Zhao *et al*.^38^ have also reported that eight pathways overlapped between the development of these two types of diabetes. The type 1 diabetes pathway, which promoted the autoimmune destruction of pancreatic β-cells, was determined to be significantly associated with GDM^38^. The common HLA class II variants identified in our study undoubtedly add another common feature between GDM and type 1 diabetes. In addition, these variants could be used as predictive factors for the potential occurrence of postpartum type 1 diabetes amongst mothers with GDM.

Etiologic and preventive fractions are extensively used in epidemiology. The interpretation of these two values should be used cautiously due to the possibility of source of bias, such as different diagnostic criteria for GDM being used and the age distribution of patients used amongst the different studies. However, EF and PF may contribute to our understanding of the mechanism that link HLA with GDM. Amongst the HLA class II molecules, DRB1*1302 and DR17 were strongly associated with susceptibility to GDM (EF of 0.17 for DRB1*1302 and 0.15 for DR17), while DQB1*0602 and DQ6 were determined to be main protective factor against GDM (PF of 0.07 for DQB1*0602 and 0.03 for DQ6).

There are still some limitations in our study. Firstly, our study only analyzed the role for HLA class II, the main susceptible variant of type 1 diabetes, in disease risk or resistance to GDM. The underlying pathogenetic mechanism common to both GDM and type 1 diabetes could be better understood if additional genetic links could be discovered. Secondly, we need to investigate more new publications when available on some of the variants that we have analyzed to generate a more robust assessment. This is because a small sample size would reduce the capacity to identify other GDM linked variants. For instance, the protective association that we identified between DQB1*0203 allele and GDM was conducted in two studies^16^,^28^, with one of them reporting a positive association but not in the other. However, a definitive conclusion could be drawn if more data were available. Thirdly, there were a low number of selected studies used in our assessment of some of the alleles, so consequently a funnel plot analysis could not be performed on them. This again indicates that we need to further increase the size of the association studies. Hence, we should keep these limitations in mind when interpreting our present study.

In sum, our meta-analysis indicates that DQB1*02 and DRB1*1302 are firmly associated with increased risk of developing GDM, while DQB1*0602 acts in a protective role against GDM. However, these associations should be interpreted with caution and the role of HLA genes in GDM pathogenesis needs further functional investigations.

## Materials and Methods

### Data base source and search

All of the literatures that were have used to investigate the relationships between HLA class II variants and GDM were extracted from PubMed, Embase, Web of Science and China National Knowledge Infrastructure (CNKI) using the search terms “gestational diabetes mellitus” and “HLA”. The publications used in our analysis were dated up to July 1, 2016. We only selected relevant literatures published in English and Chinese for analysis. Moreover, the references of all the selected articles were manually and independently searched by two of our researchers (G.C-C and J.Y-M). If more than one article was published on the same population, we selected the most complete and updated publication for analysis. We performed meta-analysis on polymorphisms that have been examined in at least two populations.

### Selection of literatures for analysis

The publications that we have selected in our study had to meet the following criteria before inclusion: (1) relevant HLA class II variant polymorphism and GDM risk, (2) odds ratio (OR) and 95% confidence interval (CI) were presented or they could be calculated from the publication, and (3) case-control studies written in either English or Chinese. We excluded publications that had the following criteria: (1) review papers, family pedigree studies and animal studies, (2) studies that contained a lack of data, and (3) studies that did not present the target alleles.

### Data Extraction and Quality Assessment

We used two investigators (G.C.-C. and J.Y.-M.) to independently extract data from the literature database according to the selection criteria described above. All disagreements within the extracted data were resolved by a senior investigator (J.C.-X.). The following information was extracted from the publications: (1) the first author’s surname, (2) year of publication, (3) study population, (4) typing method of HLA variants, (5) diagnostic criteria of GDM, (6) number of cases and control group, and (7) study design. The HLA-DR and HLA-DQ genotypic data were grouped into serological types according to agreements from the 13th International Histocompatibility Workshop and Conference^39^. We contacted the authors of our selected studies for any additional data when necessary.

The quality of the selected publications for our analysis was assessed according to the Newcastle-Ottawa Quality Assessment Scale^40^. The system was divided into three domains with the highest score of 9 points: with 4 points for the selection of the study groups, 2 points for the comparability of the groups, and 3 points for the ascertainment of either the exposure or outcome of interest for the case-control studies. We defined the scores for 0–3, 4–6 and 7–9 as low, moderate and high quality of the publications, respectively.

### Data Generation and Analysis

Meta-analysis was conducted on all available data of polymorphisms from HLA class II variants with GDM risk using Comprehensive Meta Analysis software version 2.2.064 (Biostat Inc, NJ, USA). The statistical significance of the pooled OR was determined by Z-test. Unless otherwise stated, a *P*-value of <0.05 was considered to be nominally significant. Results were adjusted for multiple testing using the Bonferroni correction, which deflates the reported *P*-value to take into account the number of tests performed, using the formula 1 − (1 − α)^1/n^ (where α equals 0.05 and n equals the number of tests performed)^41^. We assessed the heterogeneity between studies using *P*_*h*_ and *I*^*2* ^42. If the *P*_*h*_-value was more than 0.10, a fixed-effects model was selected but otherwise a random-effects model was chosen^43^. The EF and PF were also calculated to further comprehend the relationship between class II variants and GDM^44^,^45^. Potential bias in the publications selected was measured by funnel plots and Egger’s linear regression tests.

The expected statistical power was calculated using the PS Power and Sample Size Calculations Version 3.0 software (Copyright © 1997–2009 by William D. Dupont and Walton D. Plummer, Vanderbilt Biostatistics, Nashville, TN), which indicates the true association between HLA class II polymorphisms and GDM. The level of significance was set at 0.05. The ORs of each study represented the 25 and 75 percentiles of the distribution of effect sizes for HLA alleles and groups (Supplementary Tables S1 and S2).

## Additional Information

**How to cite this article**: Guo, C.-c. *et al*. The relationships between HLA class II alleles and antigens with gestational diabetes mellitus: A meta-analysis. *Sci. Rep*. **6**, 35005; doi: 10.1038/srep35005 (2016).

## Supplementary Material

Supplementary Information

## Figures and Tables

**Table 1:** Characteristics of included studies in this meta-analysis.

No	First Author	Year	Population	Typing method	Diagnostic criteria	Patients	Controls	Molecular or serological study
1	Qin^19^	2015	Chinese	PCR-SSP	ND	100	100	Molecular
2	Papadopoulou^15^	2012	Swedish	PCR-SSP	WHO criteria, 1999	452	168	Molecular
3	Papadopoulou^21^	2009	Swedish	PCR-SSP	OGTT*	764	1191	Molecular
4	Wang^27^	2008	Chinese	PCR-SSP	OGTT**	39	42	Molecular
5	Zhou^28^	2007	Chinese	PCR-SSP	OGTT**	26	42	Molecular
6	Liu^16^	2006	Chinese	PCR-SSP	OGTT**	50	50	Molecular
7	Li^29^	2005	Chinese	PCR-SSP	OGTT**	116	73	Molecular
8	Zhao^18^	2005	Chinese	PCR-SSP	WHO criteria, 1998	48	48	Molecular
9	Shaat^22^	2004	Scandinavian and Arabian	Hybridisation	OGTT*	500	550	Molecular
10	Song^23^	2002	Chinese	PCR-SSP	NDDG criteria	30	40	Molecular
11	Weng^11^	2002	Swedish	Dot-blotting	OGTT*	65	86	Molecular
12	Ferber^24^	1999	German	PCR-SSO	GDA criteria	184	254	Molecular
13	Vambergue^25^	1997	French	AFLP	Carpenter and Coustan’s criteria	95	95	Molecular
14	Acton^13^	1997	African-American	Microdroplet cytoxicity procedure and/or PCR-SSP	NDDG criteria	465	232	Molecular
15	Lapolla^17^	1996	Italian	Microlymphocytotoxicity method	Carpenter and Coustan’s criteria	52	51	Serological
16	Rubinstein^26^	1981	Puerto Rican and American	TCF	ND	136	417	Serological

*PCR* polymerase chain reaction, *SSP* sequence-specific primers, *SSO* sequence-specific oligonucleotide, *AFLP* Amplification Length Fragment Polymorphism, *TCF* two-color fluorescence, OGTT* based on 75-g OGTT and defined as a 2-h capillary glucose concentration (CGC) of at least 9 mmol/L, OGTT** based on 75-g OGTT and met at least two following conditions (0-h CGC ≥ 5.6 mmol/L; 1-h CGC ≥ 10.3 mmol/L; 2-h CGC ≥ 8.6 mmol/L; 3-h CGC ≥ 6.7 mmol/L), *NDDG* The national diabetes Data Group, *GDA* German Diabetes Association, *ND* not available data.

**Table 2:** Association between HLA DQB1 and DRB1 alleles with GDM.

HLA	Number of populations	OR (95%CI)	*P*	Adjusted *P*	*P*_*h*_	I^2^
DQB1*02	6	1.36 (1.13–1.63)	0.001	0.034	0.538	0
DQB1*0201	6	1.48 (0.89–2.47)	0.129	1	0.044	56.23
DQB1*0203	2	3.27 (1.21–8.81)	0.019	0.646	0.601	0
DQB1*0301	4	0.90 (0.55–1.47)	0.673	1	0.017	70.40
DQB1*0302	9	1.03 (0.88–1.20)	0.755	1	0.780	0
DQB1*0303	3	1.05 (0.57–1.95)	0.866	1	0.792	0
DQB1*0305	3	0.93 (0.41–2.12)	0.857	1	0.611	0
DQB1*0401	2	0.82 (0.31–2.18)	0.694	1	0.984	0
DQB1*0402	4	0.35 (0.16–0.78)	0.010	0.340	0.718	0
DQB1*0501	3	1.03 (0.63–1.71)	0.899	1	0.948	0
DQB1*0502	4	1.12 (0.57–2.19)	0.744	1	0.597	0
DQB1*0503	3	0.73 (0.29–1.82)	0.497	1	0.540	0
DQB1*0601	4	0.62 (0.29–1.30)	0.205	1	0.113	49.81
DQB1*0602	11	0.74 (0.64–0.86)	0.0001	0.0034	0.187	27.00
DQB1*0603	4	0.86 (0.68–1.08)	0.194	1	0.848	0
DQB1*0604	3	1.27 (0.59–2.74)	0.545	1	0.428	0
DRB1*01	4	0.73 (0.52–1.03)	0.075	1	0.468	0
DRB1*02	2	1.30 (0.96–1.75)	0.087	1	0.314	1.49
DRB1*03	4	1.37 (1.03–1.83)	0.031	1	0.482	0
DRB1*0301	5	3.16 (1.31–7.64)	0.011	0.374	0.012	68.84
DRB1*04	4	1.42 (0.97–2.08)	0.071	1	0.118	48.95
DRB1*0402	2	0.59 (0.21–1.67)	0.319	1	0.452	0.00
DRB1*0405	2	1.01 (0.12–8.40)	0.994	1	0.149	51.87
DRB1*07	4	1.28 (0.94–1.74)	0.117	1	0.563	0
DRB1*08	4	0.91 (0.47–1.77)	0.781	1	0.750	0
DRB1*09	4	1.11 (0.72–1.70)	0.642	1	0.414	0
DRB1*10	3	0.96 (0.29–3.22)	0.949	1	0.301	16.82
DRB1*11	3	0.81 (0.51–1.28)	0.365	1	0.805	0
DRB1*12	3	0.80 (0.51–1.26)	0.342	1	0.513	0
DRB1*13	2	0.84 (0.18–3.88)	0.819	1	0.298	7.75
DRB1*1302	3	3.37 (2.03–5.60)	2.7 × 10^−6^	9.2 × 10^−5^	0.666	0
DRB1*14	2	0.89 (0.43–1.83)	0.750	1	0.896	0
DRB1*15	2	0.60 (0.25–1.48)	0.270	1	0.138	54.48
DRB1*16	2	1.58 (0.63–3.92)	0.328	1	0.279	14.82

*OR* odds ratio, *CI* confidence interval, *P* probability tested for overall effect, *Adjusted P* corrected p-values after Bonferroni correction, *P*_*h*_ probability tested for heterogeneity of included studies.

**Table 3:** Association between HLA-DQ and -DR antigens with GDM.

HLA	Number of populations	OR (95% CI)	*P*	Adjusted *P*	*P*_*h*_	*I*^*2*^
DQ2	12	1.36 (1.10–1.67)	0.004	0.088	0.058	42.60
DQ4	4	0.50 (0.23–1.09)	0.082	1	0.982	0
DQ5	4	0.98 (0.70–1.37)	0.895	1	0.760	0
DQ6	11	0.81 (0.69–0.94)	0.008	0.176	0.743	0
DQ7	4	0.90 (0.55–1.47)	0.674	1	0.018	70.40
DQ8	10	1.02 (0.87–1.19)	0.802	1	0.840	0
DQ9	3	1.05 (0.57–1.96)	0.865	1	0.792	0
DR1	4	1.50 (0.99–2.27)	0.056	1	0.493	0
DR2	3	0.83 (0.57–1.22)	0.346	1	0.250	27.92
DR3	4	0.88 (0.41–1.88)	0.733	1	0.101	51.83
DR4	4	1.13 (0.81–1.56)	0.468	1	0.149	43.78
DR5	2	0.68 (0.34–1.38)	0.290	1	0.111	60.57
DR6	2	2.46 (0.87–6.92)	0.089	1	0.380	0
DR7	7	1.23 (0.97–1.56)	0.082	1	0.475	0
DR8	4	0.92 (0.49–1.72)	0.791	1	0.817	0
DR9	6	1.19 (0.80–1.78)	0.387	1	0.520	0
DR10	4	0.78 (0.25–2.39)	0.658	1	0.347	9.17
DR12	4	0.81 (0.52–1.26)	0.354	1	0.711	0
DR13	4	2.46 (1.02–5.90)	0.044	0.968	0.028	66.94
DR15	2	0.59 (0.27–1.31)	0.196	1	0.325	0
DR16	3	1.45 (0.64–3.33)	0.375	1	0.510	0
DR17	5	3.16 (1.31–7.64)	0.011	0.242	0.012	68.84

*OR* odds ratio, *CI* confidence interval, *P* probability tested for overall effect, *Adjusted P* corrected p-values after Bonferroni correction, *P*_*h*_ probability tested for heterogeneity of included studies.
